# Handedness and Graspability Modify Shifts of Visuospatial Attention to Near-Hand Objects

**DOI:** 10.1371/journal.pone.0170542

**Published:** 2017-01-26

**Authors:** Hayley A. Colman, Roger W. Remington, Ada Kritikos

**Affiliations:** 1 School of Psychology, University of Queensland, Brisbane, Queensland, Australia; 2 Department of Psychology, University of Minnesota, Minneapolis, Minnesota, United States of America; Universita degli Studi di Bologna, ITALY

## Abstract

We examined how factors related to the internal representation of the hands (handedness and grasping affordances) influence the distribution of visuospatial attention near the body. Left and right handed participants completed a covert visual cueing task, discriminating between two target shapes. In Experiment 1, participants responded with either their dominant or non-dominant hand. In Experiment 2, the non-responding hand was positioned below one of two target placeholders, aligned with the shoulder. In Experiment 3 the near-monitor hand was positioned under the placeholder in the opposite region of hemispace, crossed over the body midline. For Experiments 2 & 3, in blocked trials the palmar and back-of hand surfaces were directed towards the target placeholder such that targets appeared towards either the graspable or non-graspable space of the hand respectively. In Experiment 2, both left and right handers displayed larger accuracy cueing effects for targets near versus distant from the graspable space of the right hand. Right handers also displayed larger response time cueing effects for objects near the graspable versus non-graspable region of their dominant hand but not for their non-dominant hands. These effects were not evident for left-handers. In Experiment 3, for right handers, accuracy biases for near hand targets were still evident when the hand was crossed over the body midline, and reflected hand proximity but not functional orientation biases. These findings suggest that biased visuospatial attention enhances object identity discrimination near hands and that these effects are particularly enhanced for right-handers.

## Introduction

A substantial body of research has shown changes to the distribution of visuospatial attention towards objects when they are near hands. Specifically, the location of our hands, their posture, and individual differences such as handedness all have demonstrable impacts on how visual attention is distributed to objects near the body [1, 2, 3, 4, 5, 6, 7, 8]. In addition, how we plan to use our hands to act upon objects in our environment (action goals) impacts near-body visual processing [9, 10]. Typically, measuring near-hand attention confounds the goal of the action with the proximity of the hand. For example, one hand often is adjacent to the display with the distant hand responding to the target [5, 6, 7, 8], or both hands adjacent to the display responding to targets [2, 3, 4]. By contrast, studies with non-manual response measures (i.e. saccade / foot-pedal) disambiguate the action goal from manual responses, but still do not assess directly the impact of the action goal on the effects found. As a result, it is difficult to determine which elements of the action system contribute to near-hand visuospatial attention in each case. The aim of the present research was to investigate systematically how the relationship between the goal of the action and two internal states of the action system: handedness and graspability, influence the distribution of attention to objects near the body.

Handedness provides an index of the internal representation of our primary effectors, the hands. It plays an important role in determining how we use our hands to interact with objects in our environment. Right-handers as a group tend to be strongly right lateralised and use their right hand for most unimanual tasks, whereas left-handers are heterogeneous in their degree of laterality overall and may use either hand depending on circumstance [11, 12, 13]. Importantly, left and right-handers differ in the neural representation of their dominant and non-dominant hands. The degree of dominance is a reflection of this representation [14, 15]. Imaging studies have shown that the volume of motor cortex dedicated to the dominant hand is directly correlated with degree of handedness. *In vivo* recordings have shown that there is also greater synaptic connectivity in regions of motor cortex that represent the dominant hand compared with those that represent the non-dominant hand [14, 15]. Thus, handedness provides a behavioural indicator of structural and functional variations in the brain that manifest in differences in a variety of other cognitive domains and it is for these reasons that left handers are often excluded from cognitive research [14, 16, 17].

Importantly, evidence suggests that the relationship between handedness and experience is bidirectional. Frequency and /or familiarity of hand use modulates the perceptual processing of near body stimuli. For example, handedness influences which hand we use to complete an action [16, 18, 19, 20]. When we use one hand more often than the other, as we do with our dominant hand, we reinforce its representation over the non-dominant hand [14,15]. Because left-handers tend to be less lateralised than right-handers, sensorimotor representation of their dominant hand is not as substantial as in right-handers, but is also subject to changes relative to experience, such that if they use their non-dominant hand often, they strengthen its representation [15]. Moreover, behavioural evidence has shown that left- and right-handers have different kinematic profiles when using their dominant and non-dominant hand to grasp objects [20, 21, 22, 23, 24]. Further, evidence from neuro-imaging [25] confirms that such differences are subserved by different patterns of neural activation in left- and right-handers (see [26] for review).

There is recent evidence that handedness modulates basic elements of visual perception [27, 28]. Namely, target detection accuracy is increased for objects in proximity to the hands. In one study, left- and right-handed participants completed a non-speeded discrimination task (responding to the identity of a left, right or centrally aligned stimulus) while their dominant hand, non-dominant hand, both hands or no hand was adjacent to the monitor [27]. Compared with the no-hand condition, right handers displayed greater visual sensitivity for stimuli near their dominant but not their non-dominant hand. By comparison, left handers displayed similar sensitivity for their dominant and non-dominant hand rather than a mirror pattern of the right handed participants [27].

**Fig 1:**
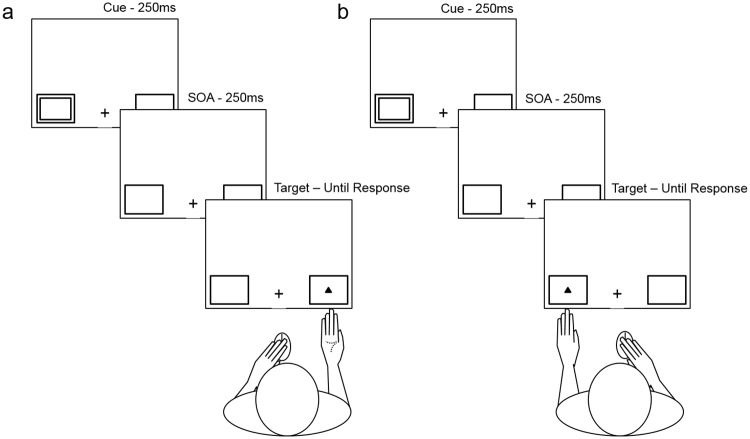
Hand postures and trial progression for Experiment 2. Fig 1A) represents the graspable posture, Fig 1B) represents the non-graspable posture. Each participant completed a block each with their monitor-adjacent hand in the A and B posture, with the posture-order counterbalanced between participants.

Other intrinsic factors such as the functional properties of hands themselves (grasping capabilities) have also been shown to influence visuospatial attention [3, 6, 7, 28]. For example, Abrams and colleagues [2] found that when hands were held to either side of a display (and responses made using display mounted response buttons) both spatial and temporal shifts in visual attention slowed compared with when responses were made by the hands distant from the display, even when hands were obscured from view. Similarly, Lloyd, et al., [6] used a covert exogenous cueing paradigm ([29] see Fig 1) to investigate the influence of hand location on attention shifting. Right handed participants held either their dominant or non-dominant hand beneath one of two target placeholders and responded via foot-pedal to target identity. The cueing effect (difference between valid and invalid RTs) was larger for targets in the hand-adjacent versus hand-distant placeholder. When the participants’ hands were crossed over the body midline and adjacent to the opposite hemispace placeholder, there were attentional biases only for targets near the right (dominant) hand and not the left. Also using an exogenous cueing task, Reed and colleagues [8] demonstrated visuospatial biases that were specific to hands and not just a result of an additive visual anchor provided by the hand. The participant’s own hand, a non-hand visual anchor or a fake hand were placed adjacent to one of two potential target locations whilst the task was performed. Targets that appeared in the hand-adjacent location were detected faster than those equidistant from fixation but distant from the hand irrespective of cue validity. Moreover, this bias was not present for the visual anchor which indicates it was specific to representation of the hand [7].

Further evidence suggests that visuospatial attention is distributed near the body, relative to both the location and grasping affordances of hands. Specifically, visual objects are detected more rapidly when near the palmar ‘grasping’ surface of the hand compared with the back-of-hand [8, 30]. To investigate this, Reed et al. [7] had participants hold their hand with either their palm or back-of-hand directed towards one of two potential target locations in a visual cueing task. Irrespective of cueing, participants were faster to detect targets appearing to their palm compared with the back-of-hand. This suggests that visuospatial attention was engaged more rapidly to the palmar (versus back) surface of the hand, because targets appearing in that location are more actable, thus reflecting an affordance bias in attention distribution [7]. Additionally, Thomas [31] found that when hands were adjacent to a visual display, precision grip postures enhanced observer performance on a form detection task (spatial sensitivity) whereas power grasps postures enhanced motion detection (temporal sensitivity).

The above findings qualify the results of earlier studies because they suggest that the orientation of the hand in relation to the target has a differential effect on the allocation of attentional resources [2, 6]. In the studies conducted by Reed, et al. [7] and Abrams, et al., [2] participants directed the palmar surface of their hand towards the screen for all conditions in which hands were held near the display. By comparison, Lloyd, et al., [6] had participants direct the back-of the hand towards the target location. Thus we do not have a clear understanding of which attentional effects are attributable to mere hand proximity and which are related to the graspable properties of the objects. This is important because each has presented evidence that the location of the hand influences different mechanisms of visuospatial attention (engagement, shifting and spatial coding). For example, Abrams et al. [2] showed that both spatial and temporal shifts in visual attention were slowed amongst visual objects near the hands. Reed et al., [7, 8] demonstrated speeded engagement and Lloyd et al., [6] found slower disengagement of attention for targets appearing near versus distant from the hand. Due to the different postures and response measures used in each, the functional orientation of the hand may contribute differently to each of these. It is also not possible to disambiguate whether the effects found by each reflect consistent visuospatial hand biases depending on task demands or the differential influence of the graspability of the targets. Moreover all of the described studies evaluated right-handed participants so it is unclear how intrinsic representation of the hands themselves may contribute to these effects.

This highlights another important consideration for paradigms which investigate hand proximity. All of the outlined intrinsic factors influence the distribution of visuospatial attention in one manner or another. Yet in examining how attention contributes to near-body visual processing, we also need to take into account the response demands of the tasks used to evaluate these (e.g., to press a key in response to the onset of a target). Extrinsic factors such as the goal of the action (e.g., to depress a key following the appearance of a target or to discriminate the identity of an object) also determine how visual attention is distributed within near-body space. Neurophysiological and behavioural research has found that visual stimuli near the body are coded relative to action centred reference frames [9, 10, 32]. For example, Cosman and Vecera [33] found that proximity of one or both hands to a visual object resulted observers more often identifying objects as foreground figures irrespective of the presence of contextual cues suggesting the object was concave (i.e. not in the foreground). These and similar findings suggest that rather than just being delineated by mere proximity to the body, visual objects are prioritised perceptually based on how we may use our hands to act on them or within the space that they are situated.

Neurons in ventral premotor cortex selectively activate to visual stimuli relative to their proximity to the hands. These neurons display maximal activation when stimuli are on or near the hands, or critically, when they move towards them [9]. In line with this, behavioural evidence has shown that action goals modulate attentional selection. When the goal of an action is to reach from one point to another, visual inputs which fall within the zone between the start and goal location of the reach, are prioritised for attention [10]. The action itself defines the area of attentional prioritisation. For example, Tipper and colleagues [10, 32] found that response times (RTs) were slower when distractors were presented between the hand and target compared with visual objects that were near the body but not within the frame of the action [10, 32].

The aforementioned research employed either the non-adjacent hand or both hands to respond to visual objects. Thus we cannot dissociate effects attributable to the manual action goal from those attributable to internal representation of the hand, because they share a bidirectional relationship. We can examine the spatial relationship between the action goal and the proximity of the hands to the target stimulus, and how that relationship modulates visuospatial attention is distributed near the body. In the present study we did so by systematically varying the relationship between the goal of the action and the proximity of the hand to examine the added contributions of handedness, hand posture and proximity. In Experiment 1 we examined how handedness influences the distribution of visuospatial attention relative to the laterality of the response hand. Experiment 2 evaluated how the combination of hand proximity, posture and the laterality of the response hand influences the distribution of visuospatial attention relative to handedness. Experiment 3 spatially dissociated the response hand from the hand adjacent to the monitor to evaluate whether lateralised biases remap with the location of the hand in the opposite region of hemispace.

## Experiment 1

The aim of Experiment 1 was to provide a measure of baseline visuospatial biases relative to handedness. We examined how the relationship between the laterality of the response hand (left versus right) and handedness (left-handed versus right-handed) influenced the distribution of attention within a visual display, to ascertain whether any response-biases were present. Left and right-handed participants completed a Posner [34] cueing task with predictable lateral cues responding to targets with either their dominant or non-dominant hand with both hands distant from the display. Because both hands were distant from the display, hand proximity and posture should not impact the pattern of results. Thus any resultant biases in visuospatial attention may be attributable to changes in visual attention based on handedness. If handedness influences the overall distribution of visuospatial attention, we expect responses to be faster to targets when responding with their dominant versus non-dominant hand irrespective of cue validity (main effect of response hand). Alternatively, if handedness influences shifts in visuospatial attention, we expect a greater cueing effect for targets aligned with the dominant hand, when participants responding with their dominant hand. Due to the greater degree of laterality exhibited by right handers it is also possible that right-handers will show more of an effect of response hand (dominant versus non-dominant) either via faster responses overall compared with left-handers when responding to targets with the dominant hand, or interacting with cueing to alter cueing effects.

### Methods

#### Subjects

Forty-two undergraduate students (25 females; 21 left-handed and 21 right-handed by self-report) from the University of Queensland (mean age 20.48 years) completed the experiment in return for course credit. The School of Psychology Ethics Review Committee, University of Queensland, Australia approved the running of this study. Verbal consent was obtained from all participants, no written consent was required by the aforementioned committee because at no stage was any uniquely identifying information obtained from the participants. Instead after they gave consent to participate they were assigned a random numerical code to identify their data set which was not able to be linked to their identity.

#### Stimuli and procedure

Stimuli were created in Microsoft PowerPoint and presented using E-Prime 2.0 at a 50-cm viewing distance on 32-cm × 48-cm LCD colour monitors (resolution of 1,680 × 1050 pixels). Two blue placeholder rectangles (7.44 ° × 5.73°) with the far edge 24.27° from fixation were presented in the bottom corners or the monitor on a black background either side of a white fixation cross (0.5°). A white peripheral cue box (6.87° × 5.15°) appeared in one of the placeholders. Targets were solid two-dimensional yellow shapes: either a triangle or circle (1.14°), that appeared in centre of one of the placeholders, 20.96° from central fixation with spatial location (left or right) randomised across trials.

The fixation cross and placeholders appeared for 700–1000ms and remained on display when a 250ms duration cue appeared in either the left or the right placeholder. At cue offset, in replication of Lloyd et al., [6] there was a 250ms stimulus onset asynchrony (SOA). The target (yellow circle or triangle) appeared in either the same location as the cue (valid cue) or the opposite location (invalid cue). Target location was invalidly cued on 30% of trials (Fig 1a) and validly cued on 70% of trials (Fig 1b). Participants were instructed to respond as quickly and accurately as possible to the target identity (circle or triangle; equally probable) by clicking either the left or right button on the computer mouse with their index / middle finger respectively, counterbalanced across participants.

Participants completed the task in a quiet, dimly lit room with their eyes approximately 50cms from the monitor. A subset of left and right handed participants were randomly assigned to complete the task with either their left hand (11 left handed participants; 10 right-handed participants) or right hand (10 left handed participants; 11 right-handed participants) resting on the table, 40cms from the centre of the monitor. The hand not engaged in completing the task was rested in the participants lap.

Of the right handed participants who responded with their left hand; five responded to triangles with their index finger and circles with their middle finger and five with the reverse. Of the right handed participants who responded with their right hand; five responded to triangles with their index finger and circles with their middle finger and six with the reverse. Of the left handed participants who responded with their left hand; five responded to triangles with their index finger and circles with their middle finger and six completed the task with the reverse arrangement. Of the left handed participants who responded with their right hand; five responded to triangles with their index finger and circles with their middle finger and five with the reverse.

### Results and Discussion

Anticipatory RTs < 150ms (0.29%) and non-stimulus driven RTs > 1000ms, (1.29%) were also excluded (see Table 1 for summary of mean RTs and accuracy for each level of each condition).

**Table 1:** Mean RTs (in ms), with standard errors (in parentheses), percentages of accuracy, for left and right handed participants, separated by response hand (left, right), target location (left target, right target), and cue validity (valid, invalid).

Handedness	Response hand	Target location		Cue validity		Cueing effect
				Valid	Invalid	
Left	Left	Left Target	RT	549.48(20)	661.08(21)	111.59(21)
			%	.97	.92	.03
		Right Target	RT	554.16(22)	673.19(25)	119.04(20)
			%	.95	.96	.01
	Right	Left Target	RT	572.48(18)	693.94(18)	121.46(22)
			%	.95	.91	.07
		Right Target	RT	551.37(19)	719.20(15)	167.83(21)
			%	.97	.98	-.03
Right	Left	Left Target	RT	566.33(19)	663.43(23)	97.10(22)
			%	.97	.96	.01
		Right Target	RT	567.08(15)	698.90(21)	131.82(21)
			%	.97	.94	.02
	Right	Left Target	RT	576.95(28)	659.70(24)	82.74(21)
			%	.95	.90	.06
		Right Target	RT	571.58(26)	690.25(26)	118.66(20)
			%	.96	.93	.02

#### RT analysis

A mixed repeated measures ANOVA was conducted with the within subjects factor target location (left target; right target) and the between subjects factors of handedness (left handed; right handed) and response hand (left; right). Cueing effect, computed as the invalid RTs–valid RTs, was the dependant variable. The only significant finding was a main effect of target location *F*(1,38) = 10.28, *p* = .003, $\eta_{p}^{²}=.21$. There was a greater cueing effect for right versus left sided targets, irrespective of handedness and response hand. No interactions reached statistical significance.

#### Error analysis

We conducted a mixed repeated measures ANOVA with the same factors as the RT analysis. Accuracy cueing effect served as the dependant variable. This was computed as the mean accuracy for validly cued targets minus the mean accuracy invalidly cued targets. The analysis revealed a main effect of target location *F*(1,38) = 7.79, *p* = .008, $\eta_{p}^{²}=.17$. There was a larger cueing effect for left versus right targets, consistent with the changes in RT. We interpret these results with regards to both accuracy and as a likely reflection of stimulus-response compatibility effects related to the use of the computer mouse for which the more frequent usage is a left click [35, 36]. However, such stimulus- response compatibility effects are unlikely to impact systematically the pattern of findings in the experiments we subsequently conducted as both left and right handers would be equally impacted. Importantly, the above findings suggest that there was no demonstrable impact of handedness, or the laterality of the response-hand on covert exogenous attention. Thus, intrinsic differences in body representation related to handedness and hand laterality did not likely influence how attention was distributed/ shifted when performing a distal task.

## Experiment 2

Experiment 1 rules out any effect of handedness on the distribution of visuospatial attention in a covert orienting paradigm. Following from this, the aim of the second experiment was to systematically the combined influence of handedness, hand proximity and functional orientation of the hand (i.e. whether the palm or back-of-hand was oriented towards the display) on the distribution of visuospatial attention. We adopted posture elements from the methodologies of Lloyd et al.,[6] and Reed et al., [7] presenting the palmar (grasping) and back-of-hand (non-grasping) surfaces towards one of two target placeholders (in blocked trials) whilst keeping the hand location constant across postures. The participant’s hand was positioned below the monitor directly under one of the two target placeholders with either the palmar or back-of-hand surface oriented upwards (towards the target placeholder). Thus when targets appeared in the hand-adjacent location they were either in graspable or non-graspable space of the hand. Participants held either their dominant or non-dominant hand directly under the placeholder that corresponded with the hand side (e.g., right hand place under the right placeholder) and we compared performance between left and right-handed participants.

In this experiment, we aimed to establish which is perceptually important as a cue for attention: the relationship between the hand near the display and the target, or the relationship between response hand and the target or any combination of these. We predicted that the proximal hand would be weighted as the stronger cue for visuospatial attention. If this is the case there should be evidence of attentional prioritisation (faster RTs and improved accuracy) for targets near versus distant from the hand, more so when the grasping versus non-grasping region of the hand is oriented towards the display, as a reflection of attentional prioritisation relative to grasping affordances. Moreover, these effects should be more pronounced (in the form of greater cueing effects) when the proximal hand is dominant compared with non-dominant, as a result of greater structural and functional representation [14]. Alternatively, if the goal of the action is the primary driver for visual attention, changes to visuospatial attention should be evident when the dominant hand is responding.

### Methods

#### Subjects

Forty-six undergraduate students (35 females; 22 left-handed and 24 right-handed by self-report) from the University of Queensland (mean age 19.79 years) completed the experiment in return for course credit, all gave informed consent.

#### Stimuli and procedure

These were identical to those of Experiment 1 with the exception of the following. Of the right handed participants, 11 responded to triangles with their index finger and circles with their middle finger and 13 with the reverse. Of the left handed participants; 11 responded to triangles with their index finger and circles with their middle finger and 11 completed the task with the reverse arrangement. Participants completed two blocks of 128 trials (256 total) one with the hand graspable (Fig 1a) and one with the hand non-graspable (Fig 1b) with block order counterbalanced across participants.

Half of the participants positioned their left hand under the left bottom corner of the screen and responded to targets with their right hand (left hand proximal) and the other half positioned their right hand under the right bottom corner of the screen and responded to targets with their left hand (right hand proximal). Postures and time-courses are shown in Fig 1. In the grasping condition, the hand was positioned directly under one of the target placeholders with the palm oriented towards the placeholder (Fig 1a). In the non-grasping condition, the hand was positioned directly under one of the target placeholders with the back of the hand oriented towards the placeholder (Fig 1b).

### Results and Discussion

Participants with overall accuracy < 70% (2 participants, both right-handed) were excluded from further analyses. This resulted in 11 left-handed and 11 right handed participants completing the task with their left hand adjacent to the display and 11 left-handed and 10 right-handed participants completing the task with their right hand adjacent to the display. Anticipatory RTs < 150ms (0.13%) and non-stimulus driven RTs > 1000ms, (5.98%) were also excluded (see Table 2 for summary of mean RTs and accuracy for each level of each condition). All follow-up comparisons to interactions were evaluated against a Bonferroni corrected p-value based on the number of comparisons conducted.

**Table 2:** Mean RTs (in ms), with standard errors (in parentheses), percentages of accuracy, for left and right handed participants, separated by hand side (left, right), target location (hand distant, hand adjacent), hand posture (non-graspable, graspable), cue validity (valid, invalid) and mean cueing effect.

Experiment	Handedness	Hand side	Hand posture	Target location		Cue validity		Cueing effect
						Valid	Invalid	
Experiment 2	Left	Left	Non-graspable	Distant	RT	541.41(29)	615.38(23)	73.98(19)
					%	.95	.92	-.02
				Adjacent	RT	532.95(31)	639,41(36)	106.47(20)
					%	.95	.91	-.04
			Graspable	Distant	RT	534.61(20)	638.99(28)	104.38(20)
					%	.94	.96	-.02
				Adjacent	RT	529.52(21)	625.43(27)	95.91(23)
					%	.94	.95	-.01
		Right	Non-graspable	Distant	RT	537.79(29)	659.94(33)	122.15(20)
					%	.95	.93	-.02
				Adjacent	RT	540.55(31)	639.80(32)	99.24(25)
					%	.96	.94	.03
			Graspable	Distant	RT	503.67(23)	601.18(30)	97.52(27)
					%	.96	.94	-.01
				Adjacent	RT	498.31(24)	608.79(30)	110.48(26)
					%	.95	.85	-.10-
	Right	Left	Non-graspable	Distant	RT	566,8555(29)	645.87(23)	79.02(19)
					%	.93	.90	-.03
				Adjacent	RT	580.77(31)	649.50(36)	68.74(20)
					%	.92	.89	.04
			Graspable	Distant	RT	542.70(20)	654.96(28)	112.26(20)
					%	.91	.90	.01
				Adjacent	RT	547.51(21)	645.36(27)	97.80(22)
					%	.91	.89	.04
		Right	Non-graspable	Distant	RT	567.85(32)	714.83(36)	146.99(22)
					%	.96	.97	.01
				Adjacent	RT	568.30(34)	690.72(35)	122.42(28)
					%	.96	.97	-.01
			Graspable	Distant	RT	548.14(25)	652.74(33)	104.61(29)
					%	.97	.97	-.00
				Adjacent	RT	536.47(26)	701.87(32)	165.41(28)
					%	.97	.80	.18
Experiment 3	Left	Left	Non-graspable	Distant	RT	620.04(32)	735.31(47)	97.51(26)
					%	.98	.94	.03
				Adjacent	RT	628.57(33)	703.88(38)	116.40(35)
					%	.97	.96	.01
			Graspable	Distant	RT	596.32(28)	693.83(38)	115.28(29)
					%	.95	.96	-.01
				Adjacent	RT	597.66(30)	714.05(43)	75.31(25)
					%	.95	.98	-.02
		Right	Non-graspable	Distant	RT	591.81(45)	684.20(58)	38.81(26)
					%	.92	.88	.04
				Adjacent	RT	589.54(43)	671.33(47)	92.38(30)
					%	.94	.96	-.01
			Graspable	Distant	RT	625.39(42)	688.52(45)	81.79(31)
					%	.93	.88	.05
				Adjacent	RT	615.49(40)	654.29(45)	63.12(30)
					%	.96	.96	-.00
	Right	Left	Non-graspable	Distant	RT	553.27(30)	610.92(44)	95.26(24)
					%	.97	.95	.02
				Adjacent	RT	535.90(31)	613.77(36)	70.50(33)
					%	.98	.96	.02
			Graspable	Distant	RT	539.60(26)	634.86(36)	57.65(27)
					%	.97	.98	-.00
				Adjacent	RT	543.60(29)	614.10(40)	77.88(24)
					%	.97	.96	.01
		Right	Non-graspable	Distant	RT	580.41(45)	660.18(58)	70.33(26)
					%	.94	.93	.02
				Adjacent	RT	565.85(44)	666.55(47)	79.77(30)
					%	.96	.94	.02
			Graspable	Distant	RT	580.56(42)	659.87(45)	100.70(31)
					%	.95	.96	-.00
				Adjacent	RT	567.91(40)	638.24(45)	79.31(30)
					%	.98	.92	.05

#### RT analysis

A mixed repeated-measures ANOVA was conducted on the mean RTs the within subjects factors of target location (hand adjacent; hand distant), hand posture (non-graspable; graspable) and the between subjects factor of handedness (left handed; right handed) and proximal hand laterality (left; right). As computed in Experiment 1, RT cueing effect was the dependant variable.

There was an interaction between hand posture, target location, and proximal hand laterality *F*(1,42) = 5.81, *p* = .020, $\eta_{p}^{²}=.12$. We conducted pairwise t-tests comparing between proximal hand laterality groups for each level of hand posture and target location and found larger cueing effects for the non-graspable hand distant condition for the right proximal hand laterality versus left proximal hand laterality *t*(42) = 2.85, *p* = .007.

To examine handedness effects directly, we conducted separate ANOVAs for each handedness group with the factors, hand posture, target location and proximal hand laterality. We found no significant differences for left handers. For right handers, there was a trending interaction between hand posture, target location and proximal hand laterality *F*(1,42) = 4.06, *p* = .057, $\eta_{p}^{²}=.17$. Follow-up planned comparisons were conducted comparing the graspable and non-graspable postures for each target location (two comparisons per handedness group). For right handed participants with the right proximal hand laterality, there was a larger cueing effect when targets appeared near versus distant from the hand when the hand was in the graspable posture *t*(20) = 2.53, *p* = .032. No other effects reached significance.

#### Error analysis

Mean error was evaluated by comparing cueing effects (valid- invalid accuracy) in a mixed ANOVA with the within subjects factors of target location (hand adjacent; hand distant) and hand posture (non-graspable; graspable) and the between subjects factor of handedness (left handed; right handed) and proximal hand laterality (left; right). There was a main effect of target location *F*(1,42) = 8.52, *p* = .006, $\eta_{p}^{²}=.17$. Cueing effects were larger for hand adjacent versus hand distant targets. There was also a main effect of hand posture *F*(1,42) = 4.91, *p* = .032, $\eta_{p}^{²}=.11$, with larger cueing effects for the graspable versus non-graspable posture, consistent with a visuospatial bias relative to grasping affordances.

An interaction between target location and hand posture clarified the above main effects *F*(1,42) = 8.00, *p* = .007, $\eta_{p}^{²}=.16$. Follow-up t-tests compared target locations between hand postures. These revealed larger cueing effects for objects adjacent to versus distant from the grasping region of the hand *t*(42) = 2.66, *p* = .001. In addition, there was also an interaction between hand posture and proximal hand laterality *F*(1,42) = 5.37, *p* = .025, $\eta_{p}^{²}=.11$. We followed this up by comparing between proximal hand laterality groups (left; right) for each hand posture. There was a greater cueing effect for the graspable posture when the right versus left hand was proximal *t*(25) = 2.49, *p* = .042.

**Fig 2:**
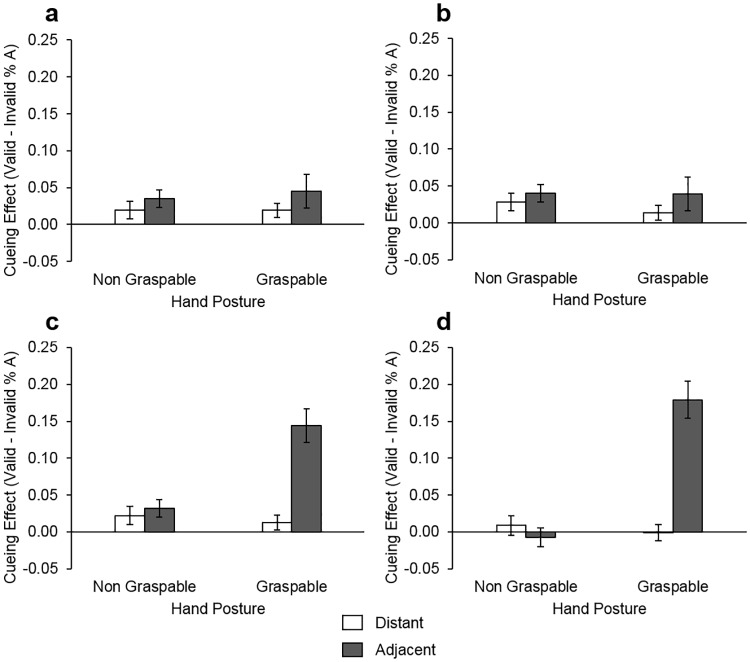
Accuracy cueing effect by target location, hand posture and handedness. Graphs (A) and (C) display mean cueing effect (with standard error bars) for left handers with their (A) left hand near and (C) right hand near. Graphs (B) and (D) display mean cueing effect (with standard error bars) for the right handers with their (B) left hand near and (D) right hand near the display.

There was also a three-way interaction between hand posture, target location and hand under the monitor *F*(1,42) = 5.93, *p* = .019, $\eta_{p}^{²}=.12$. To follow this up, we compared proximal hand laterality groups for each level of hand posture and target location (4 comparisons). Cueing effects were larger for targets adjacent to the graspable region of the hand when the right versus left hand was proximal *t*(23) = 2.49, *p* = .020 (see Fig 2 for graphical representation). Taken together, these findings suggest that there was enhanced ability to discriminate target identity, when targets onset near the graspable region of the right hand, compared with both the non-graspable region. Moreover, they indicate that there was impairment of processing for targets which onset distant from this region.

To examine effects related to handedness, we conducted separate ANOVAs with the within-subjects factors hand posture, target location and proximal hand laterality for each handedness group. As with the RT findings, we found no significant differences for left handers. For right handers there was a main effect of target location *F*(1,20) = 6.25, *p* = .021, $\eta_{p}^{²}=.24$, cueing effects were larger for hand adjacent versus hand distant targets. We also found an interaction between hand posture and target location *F*(1,20) = 4.79, *p* = .041, $\eta_{p}^{²}=.19$, however no follow-up pairwise comparisons reached statistical significance. There was an interaction between hand posture and hand-under the monitor group *F*(1,20) = 5.96, *p* = .025, $\eta_{p}^{²}=.23$. This was followed up by comparing between proximal hand laterality groups for each hand posture. There was a greater cueing effect for the non-graspable posture when the left versus right hand was under the monitor *t*(20) = 2.39, *p* = .026.

The findings of Experiment 2 indicate that there are biases in near-hand attention relative to the grasping affordances of the hand which influence the observer’s object identification accuracy. When considered in combination with the RT results, these findings suggest that visuospatial attention is biased to the grasping space of the right hand. As a result, object identification is enhanced in this location and associated impairments in identifying targets in opposite the site of biased attention. For right handers specifically, the results also suggest that there may be speeded engagement and delayed disengagement of attention to the graspable region of the dominant hand. In line with this, the results provide further evidence that left-handers may not mirror attention biases for their dominant hand in the same manner as right handers. Instead the current findings suggest that both left- and right-handers have greater accuracy in detecting objects near the graspable space of the right hand. This may reflect use-specific changes in representation, because left handers must often employ their non-dominant hand to complete tasks, due to the fact that many every-day objects (e.g., door handles) afford action from the right hand. It may alternatively reflect visuomotor biases that are specific to the left hemisphere of the brain, consistent with earlier findings [20, 21, 22]. However, it is also important to note that when broken down by handedness group, these effects dissipate which suggests that strong dominant hand grasping-space biases in right-handers may contribute to this attentional bias.

## Experiment 3

In Experiment 2, we found that the functional representation of the right hand, when proximal to the display, influenced the distribution of visuospatial attention. Accuracy of object identification was enhanced near the palm of the right hand and right handers also displayed faster engagement and delayed disengagements of attention to the grasping space of the dominant hand. The aim of Experiment 3 was to examine whether such visuospatial biases remain when the hand is crossed over the body midline, and thus are specific to functional representation of the limb.

In many instances in everyday life, we use our hands in an ipsilateral location or towards the body midline. Yet we are also capable of using hands to complete actions in the opposite region of visual hemispace; crossed over the body midline [32]. Hand-specific visuospatial biases have been interpreted by earlier research to be a reflection of the response properties of bimodal neurons [7, 8, 9, 33]. Attentional biases result from the overlapping visual and tactile representation of the space near the hand. If it is the case then the biases found in Experiment 2 should occur irrespective of the hands location in visual space. Moreover, it is important to establish whether the laterality of targets (relative to the hand) and observer handedness impact attentional distribution in space.

We used a similar methodology to Experiment 2 but the dominant or non-dominant hand under the contralateral rather than ipsilateral target placeholder. We predicted that if the visuospatial and response biases found in the second experiment were due specifically to internal representation of the right hand and not to an overall lateralisation bias, we would expect that the findings to will be replicated when the hands cross over the body midline. That is, we should find the same cueing effects for targets appearing near the right hand now in the contralateral region of visual space.

### Methods

#### Subjects

Thirty-five undergraduates (21 females; 19 right-handed; 16 left-handed by self-report) from the University of Queensland (mean age 22.14years) participated in return for course credit or for AUD10 paid remuneration, all gave informed consent.

#### Stimuli and procedure

**Fig 3:**
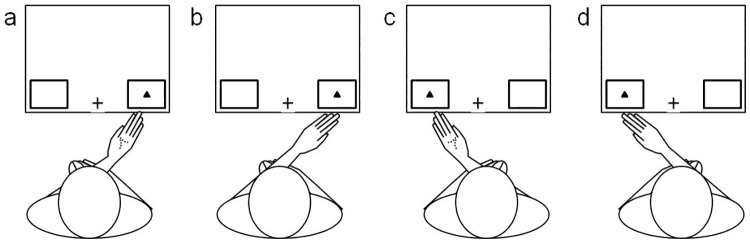
Diagram of hand postures for Experiment 2. Left hand crossed in the (a) graspable and (b) non-graspable posture and right hand crossed in the (c) graspable and (d) non-graspable posture.

Stimuli and procedure were identical to Experiment 2 with the following exceptions. Participants crossed either their right or left hand (randomised between participants) over the midline and held it under the opposite corner of the monitor (see Fig 3 for diagrammatic representation). Of the right handed participants in the left hand adjacent condition; five responded to triangles with their index finger and circles with their middle finger and five with the reverse. Of the left handed participants in the left hand adjacent condition, four responded to triangles with their index finger and circles with their middle finger and four completed the task with the reverse arrangement. Of the right handed participants in the right hand adjacent condition; five responded to triangles with their index finger and circles with their middle finger and four with the reverse. Of the left handed participants in the right hand adjacent condition, four responded to triangles with their index finger and circles with their middle finger and four completed the task with the reverse arrangement.

### Results and Discussion

Participants with overall accuracy < 70% (3 participants; all right-handed) were removed from further analyses. Anticipatory (< 150ms, < .001%) and non-stimulus driven (> 1000ms, 0.05%) RT trials were excluded from latency and accuracy analyses. Of the remaining sample, right handed and 9 left handed participants completed the task with the left hand adjacent to the monitor and 8 right handed and 8 left handed participants completing the task with their right hand adjacent to the monitor (see Table 2 for summary of mean RT and accuracy for each level of each factor). All follow-up comparisons to interactions were evaluated against a Bonferroni corrected p-value based on the number of comparisons conducted.

#### RT analysis

We conducted a mixed ANOVA with the same factors as the RT analysis for Experiment 2. There were no significant findings. To examine how handedness influenced the distribution of attention we conducted separate ANOVAs by handedness group (left; right) using cueing effect as the variable of interest. No main effects or comparisons reached statistical significance.

#### Error analysis

**Fig 4:**
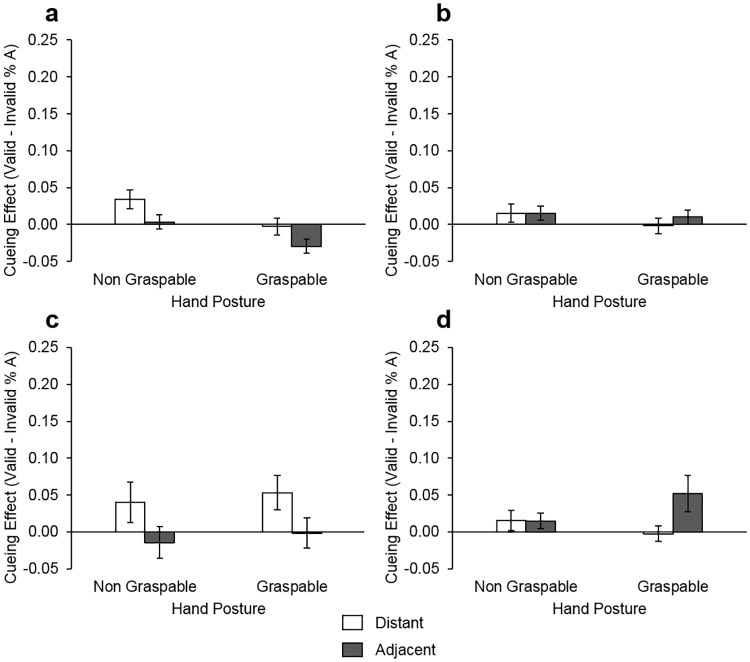
Accuracy cueing effect by target location, hand posture and handedness. Graphs (a) and (c) display mean cueing effect (with standard error bars) for left handers with their (a) left hand near and (c) right hand near. Graphs (b) and (d) display mean cueing effect (with standard error bars) for the right handers with their (b) left hand near and (d) right hand near the display.

We conducted a mixed ANOVA with the same factors as the error analysis for Experiment 2. These revealed an interaction between target location and handedness *F*(1,31) = 4.79, *p* = .036, $\eta_{p}^{²}=.13$. Follow up t-tests compared mean accuracy for each target location between handedness groups. Consistent with the results of Experiment 2, this revealed that right handers had a greater cueing effect for targets appearing adjacent to the hand, compared with those appearing distant from the hand *t*(33) = 2.50, *p* = .017. To examine how handedness influenced accuracy we conducted separate ANOVAs by handedness group (left; right) using accuracy cueing effect as the dependant variable. For left handers there was a main effect of target location *F*(1,15) = 4.81, *p* = .045, $\eta_{p}^{²}=.24$, such that there was a larger cueing effect for targets distant from versus adjacent to the hand (see Fig 4). No other effects reached significance. For right-handers there were no significant differences.

The above findings suggest that hand-centred bias in accuracy remained when hands were crossed over the body midline. This was such that object discrimination was more accurate for objects which onset near the right hand compared with those distant from it, irrespective of handedness. However, there was no longer any evidence for graspable biases for the right hand. This may be due to the reduction in the actionable value of the hand when crossed over the body midline, or to the increased difficulty of completing the task with the hand in a crossed posture. That is, the posture undertaken with the hand crossed over the body midline and the grasping side of the hand (palm) was oriented towards the display, reduces the capability for grasping. The crossed position makes grasping awkward and therefore may reduce the relevance of the hand orientation as a cue for visuospatial attention. While it is still possible to grasp an object in this posture, it is a less posturally comfortable action than the uncrossed position. Thus the affordances typically conveyed to the object near the hand by the orientation of the palm may be reduced. In turn, this may mitigate the significance of the palm orientation as a cue for attention. In addition, the results of Experiment 3 suggest that left handers no longer exhibit grasping biases for the right hand or generalised biases in the accuracy of object discrimination relative to the proximity of either the dominant or non-dominant hand, when hands are crossed over the body midline. Instead we found an exaggerated cueing effect in accuracy when targets are distant from the hand. There are a number of potential explanations for this, for example that left handers are more accurate when responding to objects in their dominant (left) side of hemispace, irrespective of hand location. However, further investigation is required to ascertain whether this is the case, as the same effect was not found in Experiment 1 where both hands were distant from the monitor.

## General Discussion

The aim of the present research was to investigate how grasping affordances and handedness work in combination with action goals to influence the distribution of visuospatial attention near hands. Experiment 1 found that when completing a distal task, exogenous cues modulated attention. Handedness or the laterality of the response hand, conversely, do not influence attention systematically. Experiment 2 found that the grasping affordances of the right hand, when proximal to a visual display, biased shifts in visuospatial attention. Both left and right handers displayed greater accuracy costs when detecting objects near versus distant from the palm of the right hand. In addition, right handers displayed more rapid engagement slower disengagement of visuospatial attention to hand-adjacent targets near the graspable region of their dominant hand. In Experiment 3, when hands were crossed over the body midline, only right-handers retained dominant hand biases in the accuracy of target identification, and grasping space biases were no longer apparent. Taken together, these findings provide evidence that visuospatial attention is distributed to objects near the hands, relative to grasping affordances, and the strength of the underlying representation.

The current research presents a number of novel findings relating to the distribution of visuospatial attention near the body. Foremost, we extended upon the findings of Reed and colleagues [8] by showing that when the task requires a higher-order aspect of visual processing (shape discrimination versus onset detection), hand location and posture modulated shifts of attention. Importantly we also illustrated that such attentional biases enhanced object identification at the site of the attention shift, and impaired object identification in the unattended zone.

The aforementioned changes in visuospatial attention run somewhat counter to the overall biases in the distribution of visuospatial attention found by Reed and colleagues [8]. Those authors found that observers were faster to detect objects near the palm of their hands, irrespective of cue validity and hand laterality [8]. We propose that this is likely the result of the discrimination (as opposed to detection) task employed in the present research. With regards to task demands, the difference between target detection and discrimination is that the latter requires focal attention whereas the former does not [37]. Specifically, object shape discrimination requires a higher order judgement of object properties than detection does, and as a result great attentional resources to discern target identity. Thus, the present findings likely reflect that internal representation of hands relative to grasping capability enhances focal attention which in turn facilitates object processing. This is a critical finding because it speaks to the level of attentional processing that is affected by the functional properties of the hands. When considered in combination with the findings of Reed et al.[8], detection of visual objects may be speeded when they are adjacent to the hands, but there are additive attentional benefits to processing object properties when they are graspable.

Our results support the proposal by Lloyd and colleagues [6] that right-lateralised biases in attention shifting evident in their research, were potentially the result of hand dominance in their sample. We extended on these by investigating a left-handed sample and found that left handers do not display the mirror attentional biases for their dominant hand that right-handers do. Instead we showed that left-handers also display biases in visuospatial attention relative to the location and functional orientation of the right hand. Also, we found that visuospatial biases for right-handed subjects are restricted to the dominant (right) hand; they did not affect performance when the non-dominant hand was proximal to the display. The present findings further clarify those of Lloyd and colleagues [6] because they show that shifts of attention occur relative to the grasping affordances of the right hand, when ipsilateral versus contralaterally aligned. Moreover, these findings fit with earlier research [20, 21, 22, 23], which has shown that visuomotor mechanisms specific to the left hemisphere of the brain have a crucial role in visually guided actions, and that individuals display similar patterns of space use, irrespective of handedness. Thus, the robust right-hand specific biases found in the present research across handedness groups may reflect an impact in attention biases that stem from or are impacted by such visuomotor biases. Stemming from this, it is important for future studies to disambiguate the left at which handedness begins to play a role in shaping or biasing the distribution of visuospatial attention, as such findings suggest that it is not as clear-cut as just a generalised bias towards faster/ more accurate detection of objects near the dominant hand.

The functional account of near-body attention posits that the allocation of additional attentional resources near the hands serves to enhance the cognitive processing of action-relevant stimulus properties [1, 2, 7, 8]. These in turn are thought to guide sensorimotor transformations required to act on hand-adjacent objects [38, 39]. We extended upon this to show that biases in near-hand attention were associated with enhancements in object identification, to the detriment of locations distant from the hand which supports the improved perception for action hypothesis. Thus, the current findings provide a precise picture of the visuospatial attention biases by illustrating that they are not only relative to handedness but more to grasping affordances. Furthermore, visuospatial biases are likely the result of expertise-dependant changes to visual perception rather than solely a reflection of strengthened right hand representation in right-dominant people. When considered in combination, the findings of Experiment 2 and 3 suggests that graspable biases in visuospatial attention do not occur in contexts where the orientation of the hand in relation to the stimulus makes grasping awkward. Yet one element of affordances not addressed in the present research, is how object affordances themselves may impact such grasping space biases. In the present study we utilised two-dimensional objects in our experimental paradigm which do not optimally elicit shape specific grasping affordances compared with three dimensional objects. As we most often interact with three dimensional objects in everyday life, it is an important direction for further study to establish how the shapes of objects themselves modulate hand-centred biases in visuospatial attention.

Following from the above, we have shown that the underlying limb representation is not the sole factor driving near-hand attention. The majority of near-hand attention research has focused primarily on right handers. The current findings contribute to understanding of body representation by demonstrating that mirrored biases are not displayed by left handers. Instead we found evidence for right-hand grasping space biases and no evidence for dominant hand associated biases in visuospatial attention for left handers. One possible explanation for these effects is that left handers are more heterogeneous in terms of the degree of hand laterality compared with right handers [14, 16]. Thus it may be the case that the lack of significant differences found in the present study between the two groups is a reflection of this heterogeneity. Moreover, the current findings, particularly with regards to Experiment 2, may reflect use-specific changes in visuospatial attention. Because left handers exist in a world designed to afford actions for the right hand, this may as a result have enhanced the underlying representation of right-grasping affordances, as was evident in the present study.

Moreover, it is also likely that left handers exhibit changes to visuospatial attention based on the functional properties and proximity of their hands but that these do not impact the mechanisms of attention probed in the present task paradigm. Earlier evidence provides support for lower level perceptual differences in the representation of the dominant and non-dominant hand between left and right handers. For example, Le Bigot and Grosjean [27] demonstrated that left handers display greater visual sensitivity (*d* ‘) for objects near both their dominant and non-dominant hand. When considered in combination with the present findings, this indicates that differences in visuospatial attention may stem from or be contributed to by underlying changes in visual sensitivity. Thus it is important for future research to establish which mechanisms of visual perception and visuospatial attention are impacted by left-hand dominance, because they appear to be less lateralised than those evident for right handers. In addition to this, the present study only investigated handedness as a discrete construct: left or right handed. Of course, a large body of previous research has shown that people exist upon a continuum when it comes to degree of overall laterality. In line with this, future research should evaluate the extent to which degree of laterality (i.e. the degree to which one is either left or right handed) impacts the attentional mechanisms evaluated in the present study.

To sum, the current study has shown that intrinsic representation of hands, both with regards to the strength of underlying representation and actable properties, modifies the distribution of visuospatial attention near the body. Our results suggest that there are biases in visuospatial attention relative to the grasping properties of hands which serve to enhance the identification of objects in this space. We have also shown that this occurs to the detriment of perceptual processing of objects which appear near to the body, but distant from the hand. These findings extend current understanding of near body attention because they demonstrate that both proximity and functional orientation of the hands play critical roles in directing visuospatial attention. Thus future research regarding near-hand attention and perception must account for the impact of both on effects of interest.

## References

[1] Abrams RA, Weidler BJ. Trade-offs in visual processing for stimuli near the hands. Atten Percept Psychophys. 2014 2 1;76(2):383–90. 10.3758/s13414-013-0583-1 24222266

[2] Abrams RA, Davoli CC, Du F, Knapp WH, Paull D. Altered vision near the hands. Cognition. 2008 6 30;107(3):1035–47. 10.1016/j.cognition.2007.09.006 17977524

[3] Adam JJ, Bovend’Eerdt TJ, van Dooren FE, Fischer MH, Pratt J. The closer the better: hand proximity dynamically affects letter recognition accuracy. Atten Percept Psychophys. 2012 10 1;74(7):1533–8. 10.3758/s13414-012-0339-3 22777734 PMC3447143

[4] Davoli CC, Du F, Montana J, Garverick S, Abrams RA. When meaning matters, look but don’t touch: The effects of posture on reading. Mem Cognition. 2010 7 1;38(5):555–62. 10.3758/MC.38.5.555 20551336

[5] Festman Y, Adam JJ, Pratt J, Fischer MH. Both hand position and movement direction modulate visual attention. Front PsychoL. 2013;4. 10.3389/fpsyg.2013.00657 PMC3787593 24098288

[6] Lloyd DM, Azañón E, Poliakoff E. Right hand presence modulates shifts of exogenous visuospatial attention in near perihand space. Brain Cognition. 2010 7 31;73(2):102–9. 10.1016/j.bandc.2010.03.006 20403655

[7] Reed CL, Betz R, Garza JP, Roberts RJ. Grab it! Biased attention in functional hand and tool space. Atten Percept Psychophys. 2010 1 1;72(1):236–45. 10.3758/APP.72.1.236 20045892

[8] Reed CL, Grubb JD, Steele C. Hands up: attentional prioritization of space near the hand. J Exp Psychol Hum Percept Perform. 2006 2;32(1):166 10.1037/0096-1523.32.1.166 16478334

[9] Rizzolatti G, Scandolara C, Matelli M, Gentilucci M. Afferent properties of periarcuate neurons in macaque monkeys. II. Visual responses. Behav Brain Res. 1981 3 31;2(2):147–63. 7248055 10.1016/0166-4328(81)90053-x

[10] Tipper SP, Howard LA, Houghton G. Action–based mechanisms of attention. Philos T Roy Soc B.1998 8 29;353(1373):1385–93. 10.1098/rstb.1998.0292 PMC1692337 9770231

[11] Buckingham G, Main JC, Carey DP. Asymmetries in motor attention during a cued bimanual reaching task: Left and right handers compared. cortex. 2011 4 30;47(4):432–40. 10.1016/j.cortex.2009.11.003 20100609

[12] Gardner MR, Potts R. Hand dominance influences the processing of observed bodies. Brain Cognition. 2010 6 30;73(1):35–40. 10.1016/j.bandc.2010.02.002 20338681

[13] Gentilucci M, Daprati E, Gangitano M. Gentilucci M, Daprati E, Gangitano M. Right-handers and left-handers have different representations of their own hand. Cog Brain Res. 1998 1 31;6(3):185–92. 10.1016/S0926-6410(97)00034-7 9479070

[14] Aboitiz F, Scheibel AB, Fisher RS, Zaidel E. Fiber composition of the human corpus callosum. Brain Res. 1992 12 11;598(1):143–53. 1486477 10.1016/0006-8993(92)90178-c

[15] Volkmann J, Schnitzler A, Witte OW, Freund HJ. Handedness and asymmetry of hand representation in human motor cortex. J Neurophysiol 1998 4 1;79(4):2149–54.24. 9535974 10.1152/jn.1998.79.4.2149

[16] Geschwind N. The biology of cerebral dominance: Implications for cognition. Cognition. 1984 8 31;17(3):193–208. 6542838 10.1016/0010-0277(84)90006-4

[17] Sörös P, Knecht S, Imai T, Gürtler S, Lütkenhöner B, Ringelstein EB, et al Cortical asymmetries of the human somatosensory hand representation in right-and left-handers. Neurosci Lett. 1999 8 20;271(2):89–92. 10477109 10.1016/s0304-3940(99)00528-5

[18] Meegan DV, Tipper SP. Reaching into cluttered visual environments: spatial and temporal influences of distracting objects. Q J Exp Psychol—A. 1998 5. 10.1080/027249898391611 9621840

[19] Corballis MC. Laterality and myth. Am Psychol. 1980 3;35(3):284 10.1037/0003-066X.35.12.1147 7377654

[20] Gonzalez CL, Whitwell RL, Morrissey B, Ganel T, Goodale MA. Left handedness does not extend to visually guided precision grasping. Exp Brain Res. 2007 182(2), 275–279. 10.1007/s00221-007-1090-1 17717653

[21] de Bruin N, Bryant DC, Gonzalez CL. “Left neglected,” but only in far space: spatial biases in healthy participants revealed in a visually guided grasping task. Arm and Hand Movement: Front Neurol 2015 61 10.3389/fneur.2014.00004 PMC3898521 24478751

[22] Gonzalez CL, Ganel T, Goodale MA. Hemispheric specialization for the visual control of action is independent of handedness. J Neurophysiol 2006 95, 3496–3501. 10.1152/jn.01187.2005 16495359

[23] Stone KD, Gonzalez CL. Grasping with the eyes of your hands: hapsis and vision modulate hand preference. Exp Brain Res 2014 232, 385–393 10.1007/s00221-013-3746-3 24162864

[24] Stone KD, Gonzalez CLR. Grasping with the eyes of your hands: Hapsis and vision modulate hand preference. Exp Brain Res 2014 232: 385 10.1007/s00221-013-3746-3 24162864

[25] Begliomini C, De Sanctis T, Marangon M, Tarantino V, Sartori L, Miotto D, et al An investigation of the neural circuits underlying reaching and reach-to-grasp movements: from planning to execution. Front Hum Neurosci. 2014 8, 676 10.3389/fnhum.2014.00676 25228872 PMC4151344

[26] Stone KD, Gonzalez CL. The contributions of vision and haptics to reaching and grasping. Front Psychol. 2015 6. 10.3389/fpsyg.2015.01403 PMC4584943 26441777

[27] Le Bigot N, Grosjean M. Effects of handedness on visual sensitivity in perihand space. PloS one. 2012 8 1;7(8):e43150 10.1371/journal.pone.0043150 22912813 PMC3422297

[28] Holmes NP, Spence C. The body schema and multisensory representation (s) of peripersonal space. Cognitive processing. 2004 6 1;5(2):94–105. 10.1007/s10339-004-0013-3 16467906 PMC1350799

[29] Posner MI. Orienting of attention. Q J Exp Psychol. 1980 2 1;32(1):3–25. 7367577 10.1080/00335558008248231

[30] Roberts KL, Humphreys GW. Action-related objects influence the distribution of visuospatial attention. Q J Exp Psychol. 2011 4 1;64(4):669–88. 10.1080/17470218.2010.520086 21113857

[31] Thomas LE. Grasp posture alters visual processing biases near the hands. Psychol Sci. 2015 5 1;26(5):625–32. 10.1177/0956797615571418 25862545 PMC4426223

[32] Tipper SP, Lortie C, Baylis GC. Selective reaching: evidence for action-centered attention. J Exp Psychol Hum Percept Perform. 1992 11;18(4):891 1431753 10.1037//0096-1523.18.4.891

[33] Cosman JD, Vecera SP. Attention affects visual perceptual processing near the hand. Psychol Sci. 2010 8 1. 10.1177/0956797610380697 20713634

[34] Posner MI, Cohen Y. Components of visual orienting. Attention Perform. 1984 1 1;32:531–56.

[35] Peters M, Ivanoff J. Performance asymmetries in computer mouse control of right-handers, and left-handers with left-and right-handed mouse experience. J Motor Behav. 1999 3 1;31(1):86–94. 10.1080/00222899909601894 11177622

[36] Tucker M, Ellis R. On the relations between seen objects and components of potential actions. J Exp Psychol Hum Percept Perform. 1998 6;24(3):830 9627419 10.1037//0096-1523.24.3.830

[37] Sagi D, Julesz B. Detection versus discrimination of visual orientation. Perception. 1985;14:619–28. 10.1068/p130619 6535985

[38] Goodale MA. Vision and action: The control of grasping. Intellect Books; 1990.

[39] Goodale MA, Milner AD. Separate visual pathways for perception and action. Trends Neurosci. 1992 1 31;15(1):20–5. 1374953 10.1016/0166-2236(92)90344-8

